# A Highly Efficient and Simple Construction Strategy for Producing Recombinant Baculovirus *Bombyx mori* Nucleopolyhedrovirus

**DOI:** 10.1371/journal.pone.0152140

**Published:** 2016-03-23

**Authors:** Xingjian Liu, Yonglong Wei, Yinü Li, Haoyang Li, Xin Yang, Yongzhu Yi, Zhifang Zhang

**Affiliations:** 1 Biotechnology Research Institute, Chinese Academy of Agricultural Sciences, Beijing, China; 2 State Key Laboratory of Biomembrane and Membrane Biotechnology, Institute of Zoology, Chinese Academy of Sciences, Beijing, China; 3 The Sericultural Research Institute, Chinese Academy of Agricultural Sciences, Zhenjiang, Jiangsu Province, China; Ecole des Mines d'Alès, FRANCE

## Abstract

The silkworm baculovirus expression system is widely used to produce recombinant proteins. Several strategies for constructing recombinant viruses that contain foreign genes have been reported. Here, we developed a novel defective-rescue *Bm*NPV Bacmid (reBmBac) expression system. A *CopyControl* origin of replication was introduced into the viral genome to facilitate its genetic manipulation in *Escherichia coli* and to ensure the preparation of large amounts of high quality reBmBac DNA as well as high quality recombinant baculoviruses. The *ORF1629*, *cathepsin* and *chitinase* genes were partially deleted or rendered defective to improve the efficiency of recombinant baculovirus generation and the expression of foreign genes. The system was validated by the successful expression of *luciferase* reporter gene and porcine *interferon γ*. This system can be used to produce batches of recombinant baculoviruses and target proteins rapidly and efficiently in silkworms.

## Introduction

Since its inception more than 30 years ago, the baculovirus expression vector system (BEVS) has been widely used to express heterologous foreign proteins [1–3]. *Autographa californica* nucleopolyhedrovirus (*Ac*MNPV)-Sf9 and *Bombyx mori* nucleopolyhedrovirus (*Bm*NPV)-silkworm are two typical BEVSs [4–6]. The *Bm*NPV-silkworm offers several advantages in comparison with the *Ac*MNPV-Sf9 system [7].

Baculoviruses are a group of large viruses with circular double-stranded DNA genomes of 88–153 kb [8], which makes it laborious to manipulate and generate recombinant baculoviruses. Therefore, several construction strategies have been developed to increase the efficiency of recombinant baculovirus generation. To generate the recombinant *Ac*MNPV, Patel *et al*. have developed a recombinant baculovirus shuttle strategy in yeast with yeast autonomous replicating sequences (ARS) and centromere (CEN) sequences [9]. An *Ac*MNPV baculovirus shuttle vector (Bacmid) that can be manipulated in *Escherichia coli* (*E*. *coli*) as a plasmid has also been reported [10, 11]. This Bacmid is commercially available as Bac-to-Bac BEVS. And also Kitts *et al*. have developed a BacPAK6 expression system [12] in which several *Bsu*36I sites have been introduced into the *Ac*MNPV genome to allow preparation of linearized viral DNA with essential gene deficiency for cotransfection. Based on these typical strategies, several defective baculovirus expression system have been engineered. Je *et al*. have reported an *Ac*MNPV Bacmid system that lack a portion of the essential *ORF1629* gene [13]. Jones *et al*. have also reported the inactivation of this essential gene to improve baculovirus recombination [14]. Furthermore, multigene expression has been achieved by some groups [15, 16].

The *Bm*NPV, as another important baculovirus using for foreign gene expression in cells and silkworms, has been researched deeply. There are also several strategies for generation of recombinant *Bm*NPV just as *Ac*MNPV baculovirus expression system. The Bm-Bac-to-Bac system has been established by Park’s group [17]. Wu *et al*. have developed the BmBacPAK6 system [18] based on the *Ac*MNPV BacPAK6 system. Some defective baculovirus systems have also been established [19, 20]. A *Bm*NPV expression system using the mating-assisted genetically integrated cloning (MAGIC) strategy has also been developed [21].

These methods for the generation of recombinant *Ac*MNPV and *Bm*NPV have been applied for a long period. The Bac-to-Bac and BacPAK baculovirus expression systems are the most widely used among these expression systems [22].

Baculoviruses have a biphasic life cycle in which two distinct forms of the virus spread throughout tissues and among individuals, which results in two forms of virions, namely a budded virion (BV) and an occlusion derived virion (ODV) [23]. Some virus encoded proteins involved in ODV structure are not required to maintain virus cell to cell infectivity or virus replication. [24–26]. For example, the *polyhedrin* and *p10* genes are not involved in within host virus infection or replication, and thus their promoters are used to drive the expression of foreign proteins [16]. The products of some virus genes, such as *cathepsin* (*cat*) and *chitinase* (*chi*), can impede foreign gene expression, and hence foreign gene expression efficiency increases in the absence of these two genes [27–29].

In this study, we developed a novel defective-rescue *Bm*NPV Bacmid (reBmBac) expression system to provide an effective and simple strategy for constructing recombinant baculoviruses. The reBmBac was established by using convenient tools, such as phage the λ Red recombinase system, the FLP/FRT (FLP recombinase recognition target) recombinase system [30], the *rpsL-neo* counter-resistance system [31, 32] and antibiotic resistance genes. The *cat* and *chi* genes in the reBmBac genome were inactivated [29] and replaced by an *E*. *coli CopyControl* origin of replication [33] to make the baculovirus viral genome DNA easy to manipulate and prepare in *E*. *coli*. The ORF1629 gene is essential to the virus life cycle because it encodes the viral capsid-associated protein [34]. This essential gene was stably and partially deleted in *E*. *coli* to construct the defective-rescue bacmid and ensure the efficiency and purity of the recombinant baculoviruses obtained through cotransfection [12]. In addition, a general manipulation method for any gene site in the baculovirus genome was established. The reporter gene *luciferase* (*luc*) as quality control and porcine gene *interferon γ* (PoIFN-γ) were successfully expressed by using this system.

## Materials and Methods

*E*. *coli* DH10B was obtained from Invitrogen (Carlsbad, CA, USA). *E*. *coli* BW25113/pKD46 and DH10B/pCP20 were obtained from the Molecular, Cellular and Developmental Biology Department, Kline Biology Tower 830, Yale University. The pGL3-Basic vector and luciferase assay system were obtained from Promega (Madison, WI, USA). The CopyControl pCC1BAC Vector (containing *CopyControl* origin) and *E*. *coli* TransforMax EPI300 were obtained from Epicentre (Madison, WI, USA). DH10Bac/pMON7124 (carrying a *tetracycline* resistance gene), transfer vector pVL1393 and Lipofectin were obtained from Invitrogen. Low-melting agarose was obtained from Sigma-Aldrich (St. Louis, MO, USA). Anti-PoIFN-γ antibody (Catalogue Number: AB10624) was obtained from Millipore (Boston, MA, USA), and goat anti rabbit pAb-HRP (Code No. 458) was obtained from MBL (Japan). Standard recombinant porcine IFN-γ (Catalogue Number: 985-PI-050) was obtained from R&D SYSTEMS (Minnesota, MN, USA).

### Construction of the gene-targeting vector

The **pCC-chi-cat vector** (GenBank accession number: KU749549) was used to construct BmBac via homologous recombination with *Bm*NPV. Homologous targeting arms were amplified (the primers for fusion PCR, namely Bm-chi-F, Bm-chi-R, Bm-cat-F, and Bm-cat-R, are listed in S1 Table) to remove the *cathepsin* (GeneID: 1724490) and *chitinase* (GeneID: 1724489) genes and were inserted into pUC19 via *Eco*RI/*Hin*dIII digestion. A fragment that contained the *CopyControl E*. *coli* origin of replication and a *chl*^R^ gene that was purified from the pCC1BAC vector using *Sal*I digestion was located between the arms.

The **pPolh-1629 vector** (GenBank accession number: KU749550) was used to construct the defective BmBac lacking a portion of the essential *ORF1629* gene. The homologous arms were amplified (the primers for fusion PCR, namely Bm-1629-F, Bm-1629-R, Bm-polh-F and Bm-polh-R are listed in S1 Table) from the *Bm*NPV genome and inserted into the pMD18-T simple vector. The *tetracycline* resistance (*tet*^R^) gene, which served as an antibiotic selectable marker, was amplified (tet-F, tet-R) from the pMON7124 plasmid and inserted between the arms by *Xho*I digestion.

The **pRN-FRT vector** (GenBank accession number: KU749551) contained the counter-resistance gene *rpsL-neo* (GenBank: GU084141.1), which was synthesized by Genscript Corp. (Nanjing, China) and flanked by a mutant *FRT* site. The *FRT-rpsL-neo* element was inserted into the pMD18 vector.

The transfer plasmid **pVL1393-*luc*** was used to deliver the *luc+* gene into reBmBac, and it achieved rescue with a complete *ORF1629* gene via homologous recombination in vivo during cotransfection. The *luc+* gene was removed from the pGL3-Basic vector by *Bgl*II/*Xba*I digestion and was then transferred to a *Bam*HI/*Xba*I-digested pVL1393 vector. The transfer plasmid **pVL1393-Po*IFN-γ*** was used to deliver the Po*IFN-γ* gene (NCBI Reference Sequence: NM_213948.1) [35] into reBmBac and to rescue the defective viral DNA. The porcine *IFN-γ* gene was amplified (primers for PCR, IFN-F and IFN-R are listed in S1 Table) and inserted into the pVL1393 vector by *Bam*HI/*Eco*RI digestion.

### Manipulation of the baculovirus genome in *E*. *coli*

The homologous recombination mediated by phage λ red recombinase and the elimination of the *FRT*-flanked fragment by FLP recombinase were performed according to Wanner *et al*., as described briefly below [30]. For the recombination, the targeting element with homologous arms was isolated by digestion or PCR from the targeting vector and purified using gel electrophoresis. A 1 μg sample of the targeting element was transferred into electrocompetent cells (*E*. *coli* BW25113 with phage λ Red recombinase) using an ECM 630 Electro Cell Manipulator with a voltage of 15 kV/CM according to the manufacturer’s instructions (BTX Instrument Division of Genetronics, Inc.). Positive recombinant clones were selected after culture with the appropriate antibiotic. The positive clones were further screened by PCR with primers that spanned the viral genome sequence and targeted element sequence. The BmBac vector with the *rpsL-neo* gene was transferred into electrocompetent cells (*E*. *coli* DH10B with FLP recombinase) to eliminate the *FRT*-flanked fragment (the *rpsL-neo* counter-selection gene). The counter-selection gene was removed with FLP recombinase. The positive clones were selected on *str*^R^ antibiotic plates after culture. The positive clones were also further screened by PCR.

A large amount of high-quality reBmBac DNA was induced by L-arabinose [36] and prepared according to the *CopyControl* BAC cloning kit instructions (Epicentre) in *E*. *coli* DH10B. Purified DNA was resuspended in 100 μL of TE buffer, and the concentration was measured with a UV-Vis Spectrophotometer (NanoDrop 2000, Thermo).

### Cell culture and virus amplification in cells

The *Bombyx mori*-derived cell line, Bm5, was cultured in TC100 insect cell culture medium (Applichem, Darmstadt, Germany) with 10% fetal bovine serum (FBS, Gibco, USA) at 27°C according to published procedures [37]. For the manipulation of transfection or cotransfection, Bm5 cells were cultured in 6-well plates at a constant cell density of 1x10^6^ cells per well for 12 h using TC100 medium with FBS. Then cells were washed twice in TC100 medium without FBS and added with the mixture of transfection or cotransfection. After incubation for 4–6 h, the FBS were added into the medium for culture.

For virus amplification or expression in cells, the cells were infected at an MOI of 0.1 (multiplicity of infection of 0.1 p.f.u. per cell) for 1–2 h.

### Cotransfection of the transfer vector and reBmBac for recombinant virus with foreign genes

The Bm5 cells were cultured as description. For transfection, 0.5 μg of reBmBac DNA and 1–2 μg of transfer vector DNA were mixed with 6 μL of Lipofectin in a total volume of 60 μL and incubated at room temperature for 15–30 min [38]. Cotransfection was performed with the mixture according to the Lipofectin transfection instruction manual (Invitrogen). The supernatants of cells transfected with recombinant virus was collected after culturing for 5 days at 25–27°C and stored at 4°C.

### Expression of foreign genes in silkworm larvae and pupae

Fifth instar silkworm larvae or pupae were injected with recombinant viruses (approximately 10^5^ PFU) between the abdominal knobs on the backside. Silkworm larvae and pupae were reared or incubated at 25–27°C and 65% humidity for 108–120 h. Larval haemolymph was collected by cutting the prolegs, and 1-phenyl-2-thiourea was added at final concentration of 0.1 μM to prevent melanization. Larval haemolymph and pupae were stored at -20°C for subsequent assays.

A plaque assay was performed to test the recombination efficiency using conventional methods [37]. The expression level of luciferase in 50 μg of protein lysate was assayed with a Luciferase Assay kit (Promega). The amount of protein in the lysate was measured by the Bradford method [39]. The expression of recombinant porcine IFN-γ was detected by western blotting according to the Protein Blotting Guide (Bio-Rad). The antiviral activity of recombinant porcine IFNs was assayed in a GFP-reduction assay with recombinant vesicular stomatitis virus (VSV-GFP) [40].

## Results

### Constructing BmBac (*Bm*NPV shuttle vector) by inserting of the *CopyControl* origin of replication

**Fig 1:**
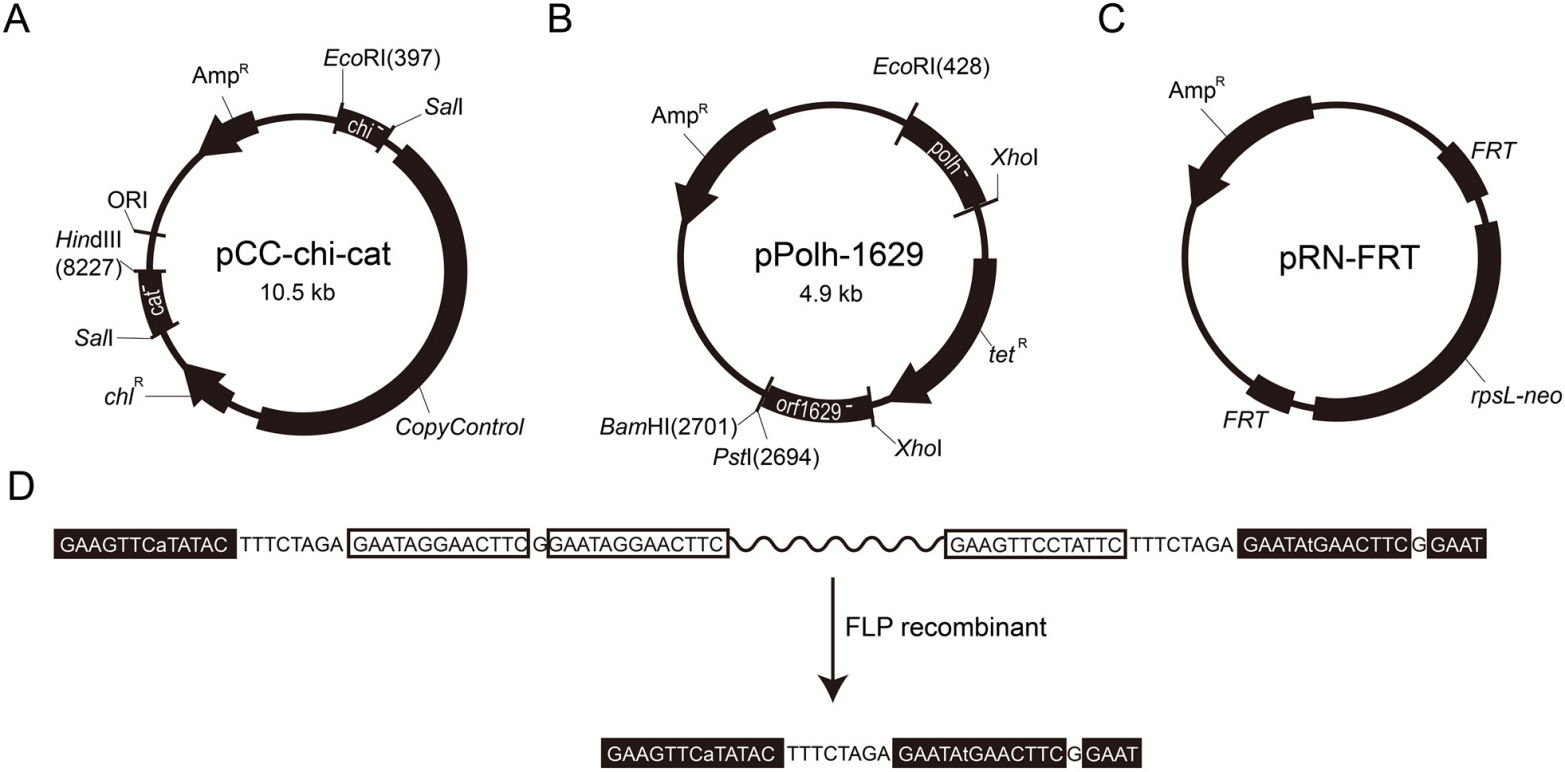
Structures of the gene-targeting vectors. (A) The pCC-chi-cat vector was used to remove the *chi* and *cat* genes and insert the *CopyControl* origin. (B) The pPolh-1629 vector was used to induce a defect in the *ORF1629*. (C) The pRN-FRT vector contained the *rpsL-neo* counter-selection gene and several mutant FRT sites. (D) FRT sequence with one mutant site was recognized and reacted using FLP recombinase. However, FRT sequences with two mutant sites were inactivated. Sequences in filled boxes are mutants, and sequences in empty boxes are wild-type.

The *CopyControl* origin of replication in *E*. *coli* (which is formed by *E*. *coli* F factor-based partitioning, a single-copy origin of replication [41] and an inducible *oriV* high-copy origin of replication [42]) was chosen to construct the *Bm*NPV shuttle vector (BmBac) which can be manipulated in *E*. *coli*. The pCC-chi-cat vector was constructed to insert this origin into the *Bm*NPV genome and replace the *cat* and *chi* genes. Homologous arms of the *cat* and *chi* genes were present in the vector. The *CopyControl* origin of replication and a chloramphenicol resistance (*chl*^R^) selectable marker gene were placed between the arms. Fig 1A shows the structure of the pCC-chi-cat vector.

**Fig 2:**
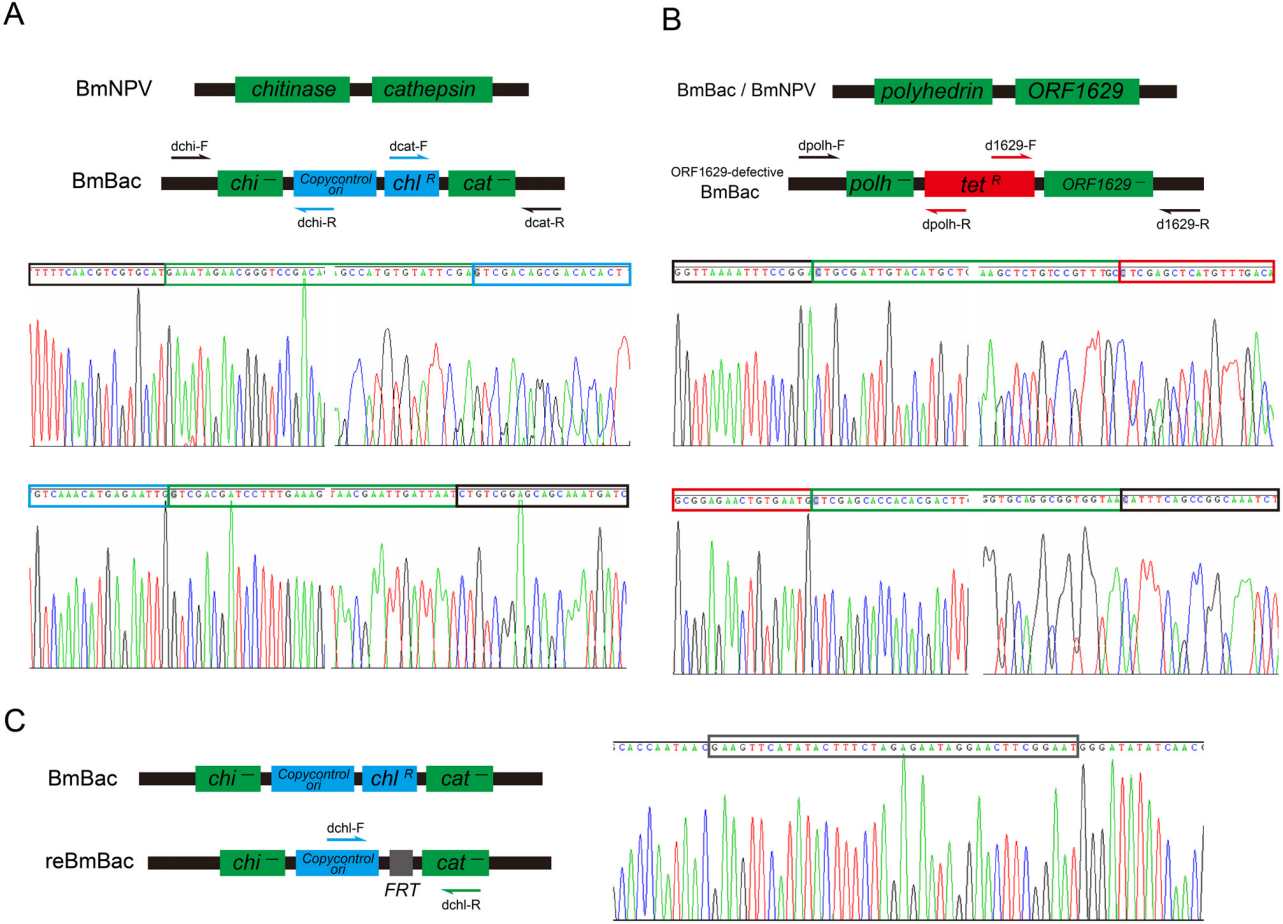
DNA sequencing of recombinant sites in BmBac and reBmBac. (A) The deletion of *chi* and *cat* genes in BmBac DNA was analyzed by PCR and sequencing as the schematic shown. (B) The defective *ORF1629* was analyzed as schematic shown. (C) The deletion of *tet*^R^ gene in reBmBac was analyzed as schematic shown. In BmBac DNA, the length of PCR product was different with that in reBmBac DNA.

The *CopyControl* origin was inserted into *Bm*NPV genomic DNA to inactivate the *cat* and *chi* genes. A 7.9 kb fragment (*chl*^R^-ori) was obtained from the pCC-chi-cat vector by *Eco*RI/*Hin*dIII digestion and was then purified using gel electrophoresis. A mixture of the purified fragment (1 μg) and *Bm*NPV DNA (2 μg) was transformed by electroporation into *E*. *coli* containing Red λ recombinase. The *chl*^R^ transformants contained a recombinant *Bm*NPV in which the *chi* and *cat* genes were deleted and replaced by the origin fragment. Positive clones were identified and screened by a PCR assay. A fragment spanning the viral genome sequence and targeting vector sequence was amplified with the primers, dchi-F, dchi-R, dcat-F and dcat-R, which are listed in S1 Table. Positive clones were further confirmed by DNA sequencing (Fig 2A). The recombinant *Bm*NPV shuttle vector was named BmBac.

BmBac DNA was extracted by the alkaline lysis method and transfected into Bm5 cells to confirm that the shuttle vector could replicate in both *E*. *coli* and insect cells. The extracted BmBac DNA was then transformed back into *E*. *coli* for the following study. The results showed that the *CopyControl* origin was successfully inserted into baculovirus DNA and that BmBac could reproduce in *Bm* cells.

The effectiveness of the *CopyControl* origin induction was examined to test the BmBac vector. Selected BmBac DNA was transformed into *E*. *coli* TransforMax EPI300, which carries an inducible *trfA* gene. L-arabinose (0.01%) was added to the *E*. *coli* host culture medium to induce DNA replication [36]. Both induced and uninduced BmBac DNA was purified and quantified using agarose gel electrophoresis. At least 5 μg of BmBac DNA was extracted from 3 mL of induced *E*. *coli* cultures, but only 0.3 μg of DNA was extracted from uninduced cultures. These results demonstrated that induction improved the DNA yield by at least tenfold.

### Construction of an *ORF1629*-defective BmBac

The *ORF1629* gene, which is located downstream of the *polyhedrin* gene, was partly deleted in our system on the basis of BacPAK and flashBAC strategies. Baculoviruses that lack a portion of the essential *ORF1629* gene cannot replicate in insect cells [12]. Therefore, the defective *ORF1629* gene can be rescued only during homologous recombination with an intact *ORF1629*, which allows the recombinant baculovirus to replicate in insect cells, when the foreign gene is inserted downstream of the *polyhedrin* gene promoter. This feature allows for more efficient acquisition of recombinant baculoviruses [12]. The pPolh-1629 vector was constructed to knock out the function of this gene. This vector contained two homologous arms of the *polyhedrin* and *ORF1629* genes, with a *tetracycline* resistance gene inserted between the arms as a selectable marker (Fig 1B).

A portion of the essential *ORF1629* gene was deleted from the BmBac DNA. A 2.3 kb *tet*^R^ element fragment that was flanked by homologous arms of the partial *polyhedrin* gene and *ORF1629* was obtained from pPolh-1629 via *Pst*I/*Eco*RI digestion and purification. DNA (1 μg) of the fragment was used to electroporate BW25113 electrocompetent cells that contained the BmBac and phage λ Red recombinase. The *tet*^R^ and *chl*^R^ transformants were selected on antibiotic-containing plates. The partial *polyhedrin* gene and *ORF1629* gene were deleted and replaced with the *tet*^R^ gene via homologous recombination. The defective BmBac was also identified and screened by a PCR assay (primers d1629-F, d1629-R, dpolh-F and dpolh-R in S1 Table) and further confirmed by DNA sequencing (Fig 2B).

BmBac DNA without an essential gene replicates in *E*. *coli* cells but not insect cells. The cotransfection of reBmBac alone was performed in Bm5 cells and showed no sign of infection. However, cotransfection of the mixture containing the above-mentioned BmBac DNA and the pVL1393 transfer vector was successful. The PCR and cotransfection results confirmed that the essential *ORF1629* gene-defective BmBac had been constructed successfully.

### Deletion of the excessive resistance gene and construction of reBmBac

We separately introduced the *chl*^R^ and *tet*^R^ genes in the *ORF1629* gene-defective BmBac vector in combination with the *CopyControl* origin of replication and partially deleted *ORF1629*. The *tet*^R^ gene would be replaced during cotransfection, and it was not present in the recombinant baculoviruses, but the *chl*^R^ gene could not be replaced. One antibiotic resistance gene is sufficient for genetic manipulation in *E*. *coli*. Thus the *chl*^R^ gene should also be deleted to ensure environmental biosafety. The strategy for deleting the *chl*^R^ gene involved the use of the FLP/*FRT* recombinase system combined with the *rpsL-neo* counter-selection marker. The *rpsL-neo* gene (GenBank: GU084141.1) flanked by *FRT* sites was synthesized and inserted into the pMD18 vector to construct the pRN-FRT vector (Fig 1C). The *FRT* sites used for this step have been optimized for high efficiency and stable knockout [43, 44]. The remaining *FRT* site was inactivated by FLP recombinase after recombination (Fig 1D).

The *FRT-rpsL-neo* element fragment was flanked by *chl*^R^ deletion homologous arms by using PCR (primers, Re-chl-F and Re-chl-R, in S1 Table) and was purified through gel electrophoresis. A 1 μg sample of the fragment was transformed into BW25113 electrocompetent cells that contained the *ORF1629*-defective BmBac and phage λ Red recombinase. The *tet*^*R*^ and *kan*^*R*^ (*kanamycin* resistance) transformants were selected on antibiotic-containing plates. The *chl*^R^ gene was replaced with the *FRT-rpsL-neo* fragment by homologous recombination. The positive plasmid was transformed into electrocompetent DH10B cells that contained FLP recombinase. The *rpsL-neo* counter-resistance gene was removed through FLP recombination, and the streptomycin antibiotic resistance of the DH10B cells was restored. Positive clones that lacked the *chl*^R^ and *rpsL-neo* genes were selected on *tet*^*R*^ and *str*^*R*^ (*streptomycin* resistance) antibiotic-containing plates. The *ORF1629* gene-defective BmBac with only one antibiotic resistance gene (*tet*^*R*^) was screened with a PCR assay (primers, dchl-F and dchl-R, in S1 Table) and further confirmed using DNA sequencing (Fig 2C). The deletion of *chi* and *cat* genes, introduction of *CopyControl* ori and the defective *ORF1629* gene were verified again by using the PCR analyze and sequencing as described before. This construct was named reBmBac (GenBank accession number: KU749552).

**Fig 3:**
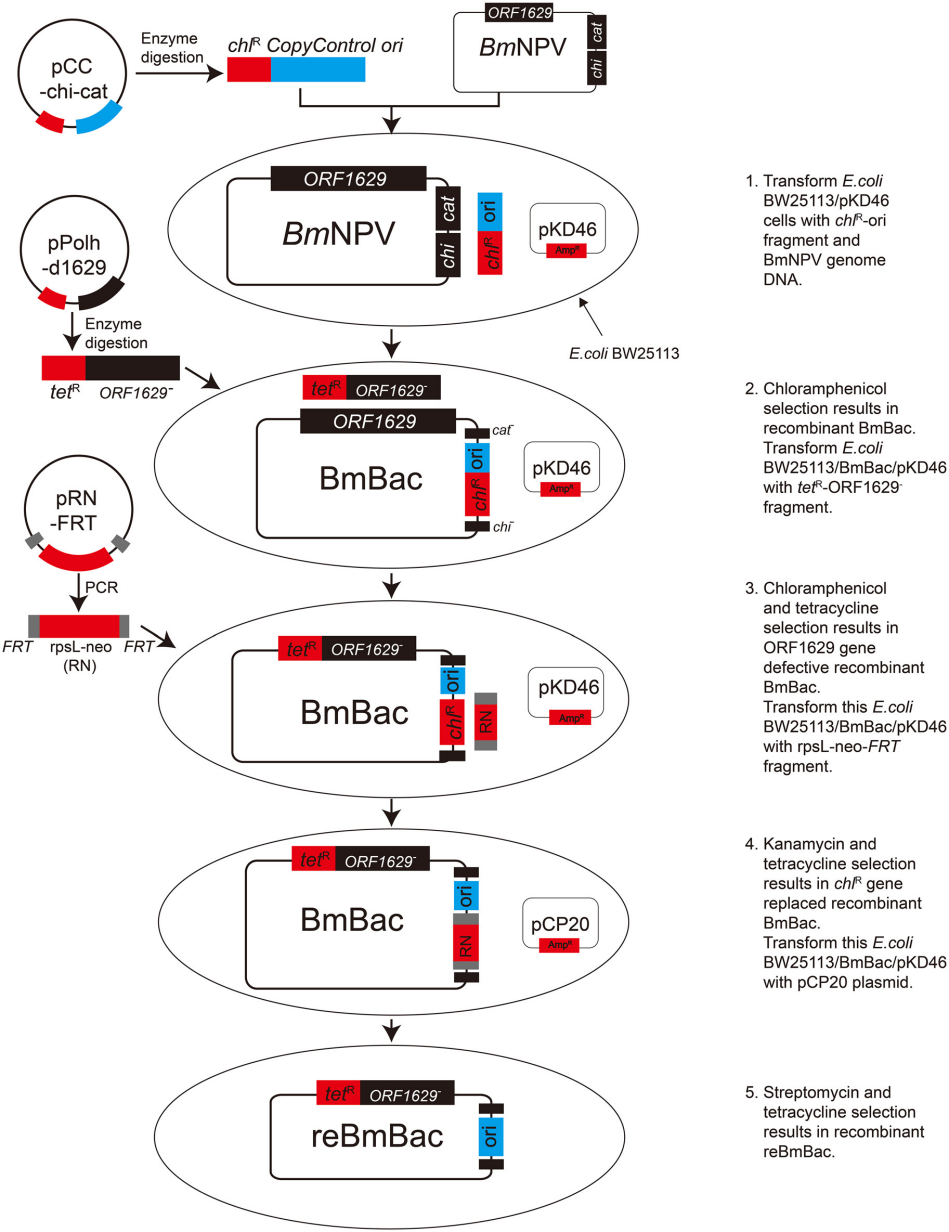
Overview of the novel reBmBac expression system (refer to Airenne *et al*.). In BmBac DNA, the *chi* and *cat* genes was replaced by the *Copycontrol* origin and a *chl*^R^ gene. Then the essential *ORF1629* gene was partially deleted by using *tet*^R^ gene. The *chl*^R^ gene was removed by using FLP/*FRT* system. The finally acquired bacmid DNA was reBmBac.

A novel reBmBac expression system was developed though the steps described above. Fig 3 illustrates an overview of this system (with reference to Airenne *et al*. [45]).

### Luciferase expression

The *luciferase* gene is one of the most sensitive reporter genes, and it is remarkably easy to detect. Therefore, this gene was used to test the reBmBac expression system. The *luciferase* gene was obtained from the pGL3-Basic vector by using *Bgl*II/*Xba*I digestion and was transferred into the *Bam*HI/*Xba*I-digested pVL1393 vector to construct the pVL1393-*luc* vector. A mixture of pVL1393-*luc* and reBmBac DNA was cotransfected into Bm5 cells. The supernatant, which contained recombinant *Bm*NPV (reBm-*luc*), was collected after 4–5 days of incubation, and it was used to infect silkworms. A 50 μg sample of protein from lysed cells or larval haemolymph was used in the luciferase assay. The luminescence of the cotransfected cells was approximately 4.86±0.47×10^6^ RLU (relative light unit), and the luminescence of silkworm larval haemolymph was approximately 3.42±0.52×10^8^ RLU. The background luminescence of the cell culture and larval haemolymph from luc-negative virus-infected samples was 150–300 RLU (S1 Fig).

We continuously amplified the recombinant reBm-*luc* from cotransfected viral stocks in Bm5 cells for three rounds, and infected silkworms to verify the purity of the recombinant baculovirus. If the recombinant *Bm*NPV is not pure, the expression levels of luciferase in cells and larvae will reduced to 10% or even less in the first two rounds of amplification. Here, the luciferase expression in silkworms was not significantly different from that of the original viral stocks. This result indicated that the stable deletion of *ORF1629* and the use of *CopyControl ori* ensured the efficiency of recombinant *Bm*NPV in cotransfection. It also showed that the viral stock acquired during cotransfection was pure to be directly used for viral amplification and protein expression. There was scarcely any wild baculovirus mixing in the target recombinant virus. S1A Fig shows the luminescence data from cells and larvae in the three rounds of amplification and expression. Therefore, the recombinant virus acquired in cotransfection was pure and with stably expression level of luciferase.

Twenty-four reBm-*luc* plaques were isolated and used to infect Bm5 cells cultured in 24-well plates to measure the recombination efficiency. Viral supernatants were collected from 24 dishes after 108–120 h of incubation. Groups of ten silkworm larvae were injected with the supernatant samples. Luciferase activity was detected in each of the 24 samples by a luminescence assay. The luminescence of 50 μg of protein from infected larval haemolymph samples that had been lysed ranged from 5×10^7^ to 5×10^8^ RLU (S1 Fig). All 24 plaques were successful recombinant *Bm*NPVs that contained the *luciferase* gene. The purity of the recombinant virus harvested via cotransfection approached 100%.

These results demonstrated that the reBmBac expression system could be used to express foreign genes successfully. The recombinant *Bm*NPV in the cotransfection viral stock could be used directly for expression.

### Expression of recombinant porcine interferon-γ

**Fig 4:**
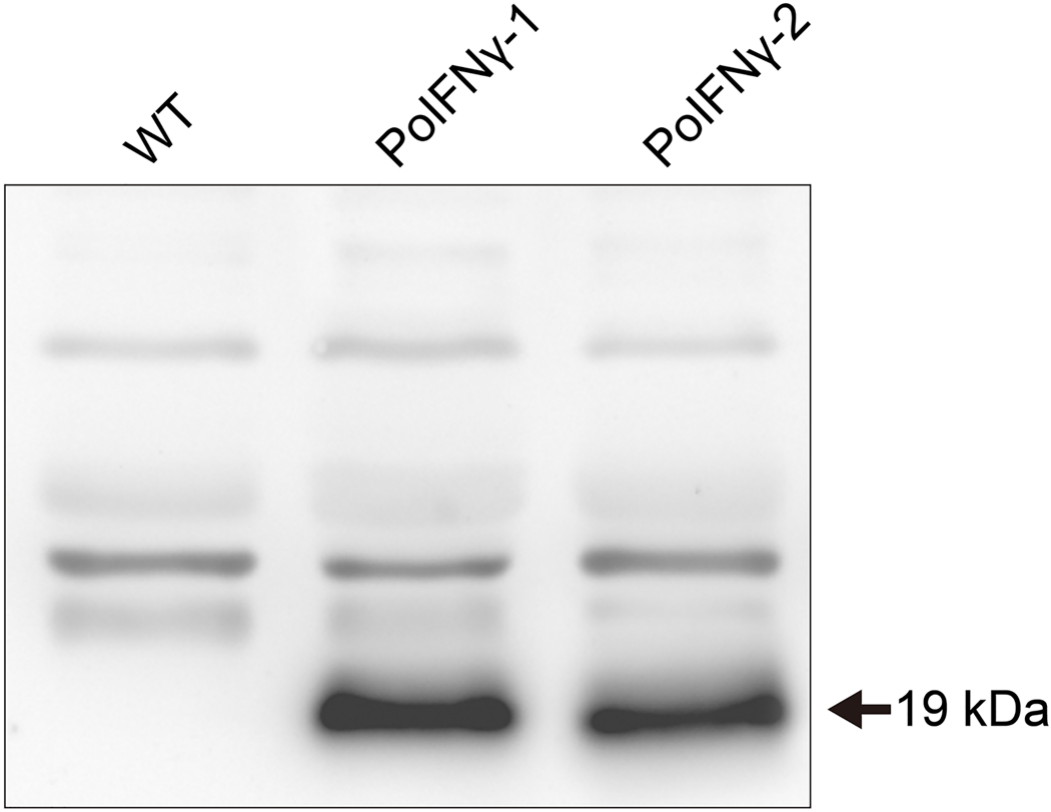
Western blot analyses of PoIFN-γ expression in silkworm larvae. The PoIFNγ-1 sample is the larval haemolymph infected with one of 24 plaques of recombinant virus stock, and the PoIFNγ-2 sample is the larval haemolymph infected with the recombinant virus acquired during cotransfection. PoIFN-γ proteins were detected in PoIFNγ-1 and PoIFNγ-2 samples as an approximately 19 kDa band. No corresponding immunoreactive protein was detected in wild-type (WT) samples.

Po*IFN-γ* gene expression was tested to further examine the utility of the reBmBac expression system. First, the Po*IFN-γ* gene was cloned into the transfer vector pVL1393. Second, the gene was inserted into the viral genome to construct recombinant reBm-Po*IFN-γ* via homologous recombination. The recombinant reBm-Po*IFN-γ* virus was injected into larvae or pupae. The expression product of porcine IFN-γ was analysed by western blotting (Fig 4) with an anti-PoIFN-γ antibody (Millipore, USA). Fig 4 shows that an approximately 19 kDa protein band that reacted with the anti-PoIFN-γ antibody was detected in the samples. No corresponding immunoreactive protein was detected in the negative control sample from larval haemolymph infected with WT *Bm*NPV. Therefore, these results indicate that porcine IFN-γ was successfully expressed in silkworms.

The antiviral activity assay indicated that the reBm-Po*IFN-γ* product exhibited antiviral activity that exceeded 1 x 10^6^ IU/mL in haemolymph. Twenty-four plaques were also isolated and used to infect silkworms. The antiviral activity assay demonstrated that the activity of these plaque samples were approximately 6.2 ± 1.3×10^5^ to 2.4 ± 0.7×10^6^ IU/mL of haemolymph (S2 Fig). The highest antiviral activity was 2.4 ± 0.7x10^6^ IU/mL, which represented a twofold improvement in antiviral activity in comparison with the cotransfected sample. The activity of Po*IFN-γ* in one mililiter of haemolymph from the best sample was equal to that of 271.4 ± 81.5 μg standard recombinant porcine IFN-γ (R&D Systems, USA).

## Discussion

**Fig 5:**
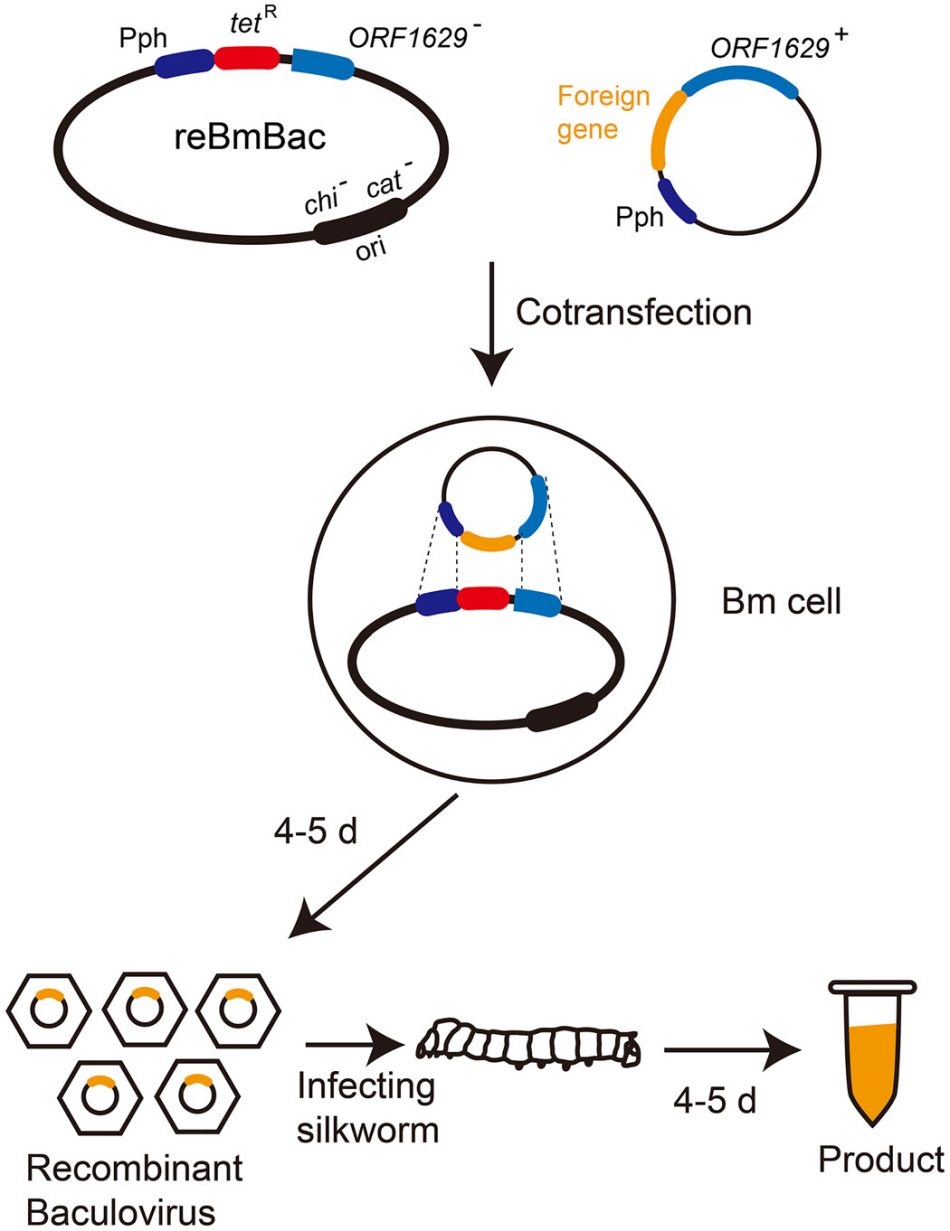
Schematic for the generation of recombinant viruses and expression of interested foreign genes. Recombinant viruses containing foreign genes were generated 4–5 days after cotransfection. The recombinant protein was harvested 4–5 days after silkworm infection.

The above data demonstrated the successful development of the novel defective-rescue reBmBac expression system. Recombinant BmNPV viral stocks were obtained within 4–5 days with this system, and recombinant proteins expressed in silkworm larvae or pupae were harvested after an additional 4–5 days (Fig 5). This system is remarkably efficient. Many experiments have shown that the expression level of a heterologous protein in five silkworm larvae or pupae is approximately equal to the expression levels of fermentation products from 1 L of sf9-*Ac*MNPV cells [7]. Therefore, this reBmBac-silkworm expression system is a rapid and highly efficient system for producing large amounts of foreign protein.

The *Bm*NPV-silkworm expression system has been reported that it is a powerful and potential system for foreign gene expression. Scientists have long attempted to develop more convenient methods for generation of recombinant *Bm*NPV and high-level expression of recombinant proteins in silkworm. The recombinant baculovirus generation strategies of *Bm*NPV are similar to those corresponding ones of *Ac*MNPV [46]. The Bac-to-Bac system and the BacPAK system are two typical and commonly used baculovirus expression system. A foreign gene can be inserted into *Bm*NPV baculovirus DNA in *E*. *coli* by using the Bm-Bac-to-Bac system, which is a convenient and easy-to-used expression vector for recombinant baculovirus construction and expression [17]. There were studies shown that the recombinant Bacmid DNA or special *E*. *coli* containing Bacmid could be used to inject the silkworm directly for expression, but the expression efficiency and level were not ideal [17, 47]. The Bac-to-Bac system is widely used in the BEVS expression fields, which benefit from the convenience and stabilization of its operation in *E*. *coli*. However, this system is also relatively inconvenient for the heterologous expression of many genes because each gene must be manipulated independently in *E*. *coli*. In the BmBacPAK system, there are several *Bsu*36I sites in baculovirus DNA [48]. The *Bsu*36I digested baculovirus DNA is linear and lacks part of *ORF1629*. Incomplete enzyme digestion dramatically reduces the ratio of recombinant virus. In comparison with these two BEVSs, the reBmBac expression system combines their advantages, i.e., convenient viral genome DNA preparation in *E*. *coli* and easy recombinant baculovirus production via cotransfection. The stable deletion of the essential ORF1629 gene in *E*. *coli* beforehand made the digestion step could be omitted and ensured the efficiency and purity of recombinant baculoviruses that were obtained by cotransfection. In addition, some disadvantages of these two BEVSs were avoided. It always takes at least two weeks to produce one recombinant baculovirus using Bac-to-Bac BEVS, whereas it takes only approximately five days when using reBmBac BEVS to produce various recombinant baculoviruses that contain different genes of interest at the same time. The batch production of different baculoviruses can be achieved conveniently via one-step cotransfection, and thus, a large amount of time is saved. It is much more convenient to prepare a large amount of high-quality parental viral genome DNA by using the reBmBac BEVS rather than the BmBacPAK system because of the *CopyControl* ori [33]. Besides, the *ORF1629*-defective Bacmid bBpGOZA expression system is another easy-to-use system [19]. Our reBmBac system has two major improvements relative to this system. First, the fragment replaced during homologous recombination in our reBmBac system is only a 1.2 kb *tet*^*R*^ gene. In contrast, the fragment in bBpGOZA is more than 6 kb, and it contains a miniF replicon and a *kan*^*R*^ gene. Second, the *CopyControl* origin in our system facilitates the large-scale preparation of high-quality reBmBac DNA. Our results over the course of many experiments indicated that the high quality of reBmBac DNA and transfer plasmid DNA are key factors for achieving good recombination results. We observed a greater than fivefold difference in the luciferase expression level and the expression levels of other proteins of interest depending on the quality of the DNA (S2 Table). The quality of well-preserved (fresh or store at -20°C) and bad-preserved (freeze-thawed for five rounds) transfer vectors are different. And the quality of parent reBmBac viral DNA, extracted from *E*. *coli* by induction or non-induction methods, are different. Over many protein expression trials in this reBmBac system, we found that when we used the same amount but different quality of pVL1393-luc transfer vector DNA to mix with the same tube of reBmBac DNA and to cotransfect cells, the luminescence from the cells and larvae could reach a tenfold difference. When we use the same amount but different quality of reBmBac DNA to mix with the same tube of well-preserved pVL1393-luc vector and to carry out the cotransfection, the luminescence from cells and larvae were at least fivefold different.

The expression tests of luciferase and PoIFN-γ revealed that the purity of the recombinant viruses acquired by cotransfection was sufficient to directly infect silkworms for expression. The amount of recombinant virus from one cotransfection was sufficient to infect at least 5,000 silkworm larvae or pupae. The expression was sufficiently high to perform functional research on the expressed protein. Furthermore, the three continuous rounds of viral reproduction demonstrated that the efficiency and purity of recombinant *Bm*NPV were sufficient for virus amplification and protein expression. The plaque assays of reBm-luc and reBm-Po*IFN-γ* showed that the reBmBac system allows 100% recombinant *Bm*NPV formation. Combining the three rounds of amplification test, the direct expression in silkworms obviates the need for further virus plaque purification assay. Therefore, the time required from expression plasmid production to harvesting a large amount recombinant protein is only 10 days, in theory. Obtaining recombinant virus requires only a simple cotransfection, which could be performed in batch mode and would be suitable for the expression of different foreign proteins after batches. In general, we performed 8–12 cotransfections in two 6-well cell culture plates (6 x 35 mm) for one batch and used the luciferase reporter to evaluate the recombination and expression efficiency in silkworms. We were confident that a group of recombinations and gene expression trial in the silkworms was successful when the luminescence of the cell lysate from the cotransfection reached 2 x 10^6^ RLU or greater, and the larval haemolymph achieved 5 x 10^7^ RLU or greater. These features are beneficial for the rapid and efficient expression of foreign proteins.

The three rounds of amplification and infection of reBm-luc in cells and silkworms showed that the luciferase expression level was stable by using this reBmBac system. We have used the uninduced reBmBac DNA to generate the luciferase gene recombinant *Bm*NPV. The luminescence reduced dozens-fold in the first round of amplification and expression in larvae, which indicated that the recombinant *Bm*NPV acquired in cotransfection is not pure and not suitable for directly expressing in larvae. The same phenomenon sometimes appears in the using of BacPAK6 system. Here, the stable expression data in the three rounds indicated that the reBm-luc acquired in cotransfection was pure and could be used for expression directly. In the plaque assay of reBm-luc, all of the screened virus stocks drive positive expression of luciferase. But the expression levels of them were steadily different. This is because of the reBm-luc acquired in cotransfection is a mixture of various types of recombinant viruses, while the screened virus stock is single clone recombinant virus in theory. Therefore, the expression level of luciferase of 24 plaque stocks were higher or lower than that of contransfection virus stock. And the plaque assay of reBm-Po*IFN-γ* also verified this result. Even so, the several virus stocks that drive higher expression levels could be selected for further researches or large-scale expression of target genes. Generally, the expression levels of these optimized recombinant virus stocks could be at least twofold higher than that of cotransfection stock. This optimization of expression level is recommended step for further large-scale producing.

The introduction of the *CopyControl* replicon facilitates the generation of large-plasmid DNA in *E*. *coli*. Wild [33] and the CopyControl BAC cloning kit instructions indicate that the copy number of the vectors, containing *CopyControl* origin, depends on the vectors’ size. The copy number for Bacmids could increase by 10- to 20-fold upon induction. The reBmBac DNA yield was similar in our system. The *oriV* origin of replication should be silent during genetic manipulations in baculovirus genome. The single-copy replicon allows for the autonomous replication and stable segregation of plasmids at a low number, which facilitates positive clone selection. The high-copy *oriV* origin of replication is induced when it is necessary to prepare Bacmid DNA for cotransfection. High-quality reBmBac DNA, which can be prepared easily on a large scale, is one of the key factors for successful homologous recombination. The above mentioned results showed that the quantity of reBmBac DNA extracted from 3 mL of overnight culture medium was sufficient for performing at least 10 cotransfections.

Recombinant *Bm*NPV that contained foreign genes contained no antibiotic resistance gene in our system, which provides improved environmental biosafety. The *tet*^R^ gene was replaced with a foreign gene during cotransfection. The *chl*^R^ gene was deleted with the FLP/*FRT* system and the *rpsL-neo* counter-resistance selection system. The deletion of the *chl*^R^ gene was also an example of the genetic manipulation of viral genes or the introduction of foreign genes into the other baculoviral genome sites in *E*. *coli*. This makes the multigene expression can also be achieved by using our reBmBac expression system as the using of other published system in silkworms [49]. The *FRT* site used here is a mutant site [43, 44]. The remaining FRT sequence is inactivated after recombination, which is favourable for ensuring the stability of the viral genome.

## Conclusions

A novel defective-rescue reBmBac expression system was successfully developed. The examples of luciferase and PoIFN-γ expression showed that foreign genes can be successfully expressed with this system. These results show that the novel reBmBac silkworm expression system is excellent and has the following advantages: (i) This system allows for simple and rapid generation of recombinant *Bm*NPVs carrying foreign genes. Because the defective *ORF1629* and the introduction of the *CopyControl* origin of replication, the purity of the recombinant virus harvested during cotransfection approaches 100%. The luciferase expression level could be used as the quality control for the cotransfection and expression efficiency of foreign genes in larvae. The amount and quality of recombinant viral stock is sufficient for various research applications. Plaque purification can be used to further improve the expression level but is not a necessary step. (ii) This system facilitates the batch generation of recombinant *Bm*NPV with various foreign genes, thus allowing for the mass production of proteins of interest. (iii) No antibiotic resistance gene is present in recombinant *Bm*NPV, and thus the biosafety of this system is ensured.

## Supporting Information

S1 Dataset Raw data of luciferase and PoIFN-γ expression. (XLSX)

S1 Fig Expression of luciferase in cells and larvae. The luminescence of 50 μg of proteins lysed from cells and larval haemolymph indicates the expression of luciferase. (A) Luciferase expression in cells that were consecutively passaged for three rounds is stable. The three rounds of expression in larval haemolymph were not obviously different from the original viral stocks. The Cells-Control and Larvae-Control were the cells and larvae samples which were infected with non-luc recombinant *Bm*NPV. (B) Luciferase expression of 24 plaques samples in silkworm was detected, and the luminescence of the best sample achieved 5.1 ± 1.0×10^8^ RLU/50 μg protein. This result indicates that the expression of foreign protein was doubled compared with the cotransfection sample. The “M-C” bar was the mock infected control sample. The “N-C” bar was the non-luciferase recombinant *Bm*NPV infected control sample.
(TIF)

S2 Fig Antiviral activity of the PoIFN-γ produced in larval haemolymph. The product of reBm-PoIFN-γ (cotransfection viral stock) exhibited antiviral activity that exceeded 1 x 10^6^ IU/mL haemolymph (“co-t” bar). 24 plaque viral stocks were screened using plaque assay and used to infect silkworms. The antiviral activity of the best sample was 2.4 ± 0.7x10^6^ IU/mL, which exhibited a twofold improvement in antiviral activity compared with the cotransfection sample. The commercial positive control (“c-1” bar) sample was the standard recombinant porcine IFN-γ (R&D Systems, USA). The standard sample was reconstituted at 50 μg/mL and its antiviral activity was about 4.5 x 10^6^ IU/mL. The negative control (“c-2” bar) sample was the non-interferon recombinant *Bm*NPV infected larvae and it shown no antiviral activity. The activity of PoIFN-γ in one milliliter haemolymph of the best sample (in our system) was equal to that of 271.4 ± 81.5 ug standard control sample.
(TIF)

S1 Table Primers List. (DOC)

S2 Table Luciferase expression levels of recombinant BmNPV producing with different quality of viral DNA or vector DNA. (DOC)
