# Supplementary figures and images for: A Highly Efficient and Simple Construction Strategy for Producing Recombinant Baculovirus Bombyx mori Nucleopolyhedrovirus
Source: PLoS One. 2016 Mar 23;11(3):e0152140. doi: 10.1371/journal.pone.0152140 (PMC4805210; doi:10.1371/journal.pone.0152140)

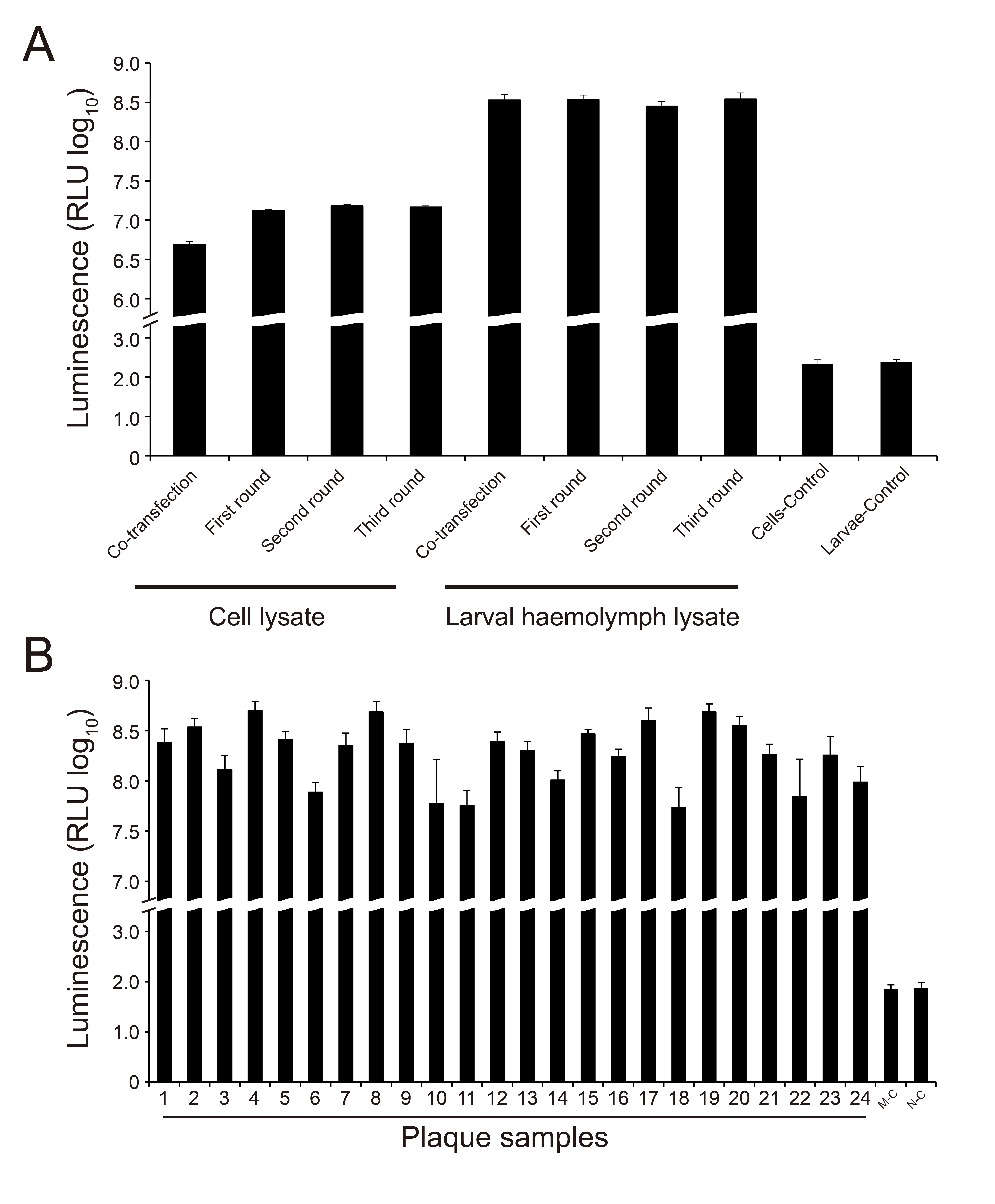

Supplement: S1 Fig — The luminescence of 50 μg of proteins lysed from cells and larval haemolymph indicates the expression of luciferase. (A) Luciferase expression in cells that were consecutively passaged for three rounds is stable. The three rounds of expression in larval haemolymph were not obviously different from the original viral stocks. The Cells-Control and Larvae-Control were the cells and larvae samples which were infected with non-luc recombinant BmNPV. (B) Luciferase expression of 24 plaques samples in silkworm was detected, and the luminescence of the best sample achieved 5.1 ± 1.0×10^8^ RLU/50 μg protein. This result indicates that the expression of foreign protein was doubled compared with the cotransfection sample. The “M-C” bar was the mock infected control sample. The “N-C” bar was the non-luciferase recombinant BmNPV infected control sample. (TIF) [file pone.0152140.s002.tif]

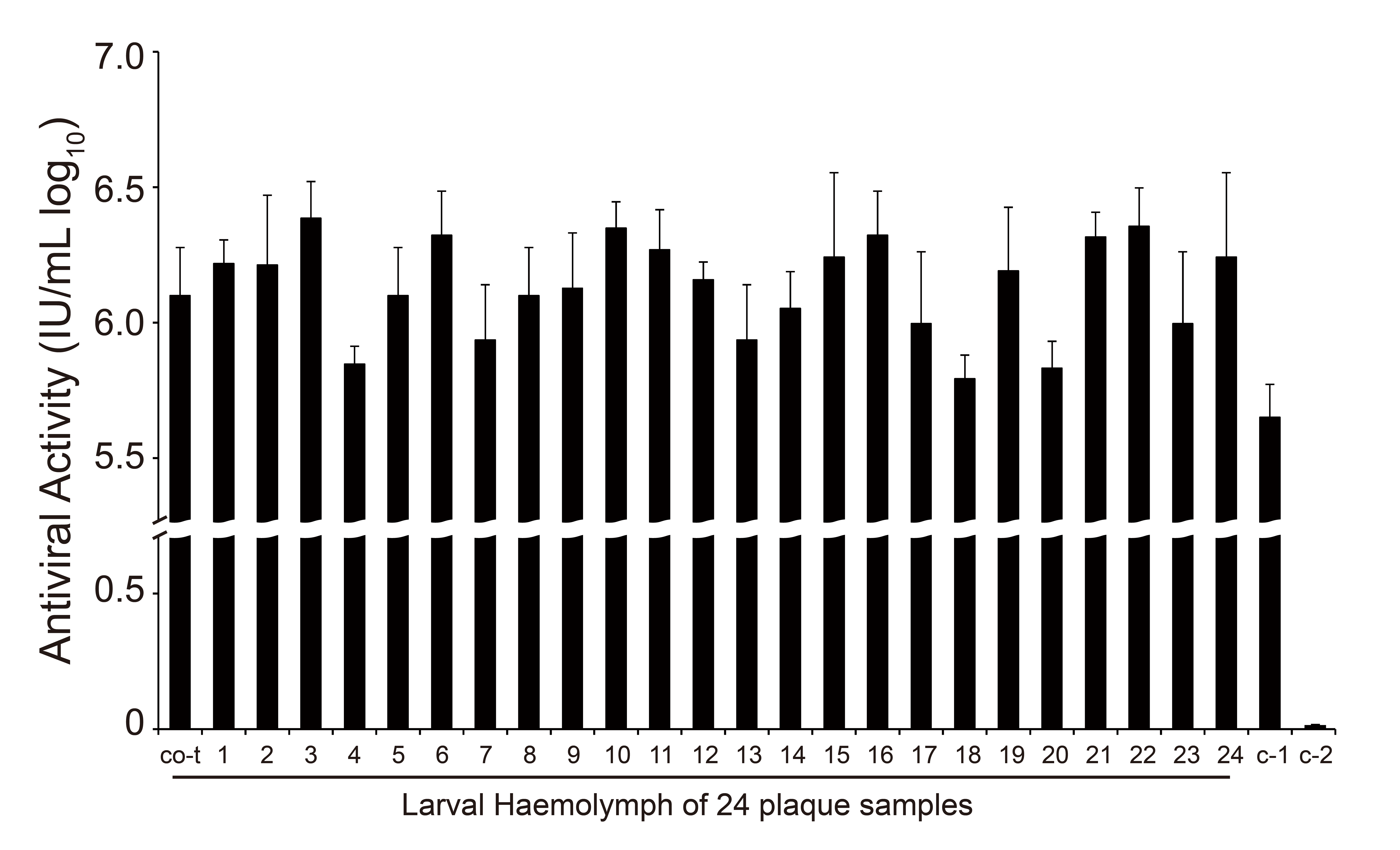

Supplement: S2 Fig — The product of reBm-PoIFN-γ (cotransfection viral stock) exhibited antiviral activity that exceeded 1 x 10^6^ IU/mL haemolymph (“co-t” bar). 24 plaque viral stocks were screened using plaque assay and used to infect silkworms. The antiviral activity of the best sample was 2.4 ± 0.7x10^6^ IU/mL, which exhibited a twofold improvement in antiviral activity compared with the cotransfection sample. The commercial positive control (“c-1” bar) sample was the standard recombinant porcine IFN-γ (R&D Systems, USA). The standard sample was reconstituted at 50 μg/mL and its antiviral activity was about 4.5 x 10^6^ IU/mL. The negative control (“c-2” bar) sample was the non-interferon recombinant BmNPV infected larvae and it shown no antiviral activity. The activity of PoIFN-γ in one milliliter haemolymph of the best sample (in our system) was equal to that of 271.4 ± 81.5 ug standard control sample. (TIF) [file pone.0152140.s003.tif]
