# Supplementary material for: A Highly Efficient and Simple Construction Strategy for Producing Recombinant Baculovirus Bombyx mori Nucleopolyhedrovirus
Source: PLoS One. 2016 Mar 23;11(3):e0152140. doi: 10.1371/journal.pone.0152140 (PMC4805210; doi:10.1371/journal.pone.0152140)
Supplement: S1 Table — (DOC) [file pone.0152140.s004.doc]

**S1 Table. Primers List.**

| Primer name | Sequence |
| --- | --- |
| Bm-chi-F | CGAATTCGAAATAGAACGGGTCCGACAG (*Eco*RI) |
| Bm-chi-R | AGGCGCTTTCAAAGGATCGTCGACTCGAATACACATGGCTGGAA (*Sal*I) |
| Bm-cat-F | TTCCAGCCATGTGTATTCGAGTCGACGATCCTTTGAAAGCGCCT (*Sal*I) |
| Bm-cat-R | CAAGCTTGATCATTTGCTGCTCCGACAG (*Hin*dIII) |
| Bm-poly-F | CGAATTCCTGCGATTGTACATGCTGTTAAC (*Eco*RI) |
| Bm-poly-R | GATTTGGCAAGTCGTGTGGTGCTCGAGGCAAACGGACAGAGCTTGTC (*Xho*I) |
| Bm-1629-F | GACAAGCTCTGTCCGTTTGCCTCGAGCACCACACGACTTGCCAAATC (*Xho*I) |
| Bm-1629-R | CGGATCCCTGCAGAGTAGATTTGCCGGCTGAAATG (*Bam*HI, *Pst*I) |
| tet-F | CCTCGAGCTCATGTTTGACAGCTTATC (*Xho*I) |
| tet-R | CCTCGAGCATTCACAGTTCTCCGCAAG (*Xho*I) |
| IFN-F | GGGATCCAACATGAGTTATACAACTTATTTCTTAG (*Bam*HI) |
| IFN-R | CGAATTCTTATTTTGATGCTCTCTGGCC (*Eco*RI) |
| Re-chl-F | CATCCGCTTATTATCACTTATTCAGGCGTAGCAACCAGGCGTTTAAGGGCACCAATAACGAAGTTCATATACTTTCTAGA (Homologous Arm) |
| Re-chl-R | AAGGAAGCTAAAATGGAGAAAAAAATCACTGGATATACCACCGTTGATATATCCCATTCCGAAGTTCATACATATTCTCTA (Homologous Arm) |
| dchi-F | GTAGACTGTTGTTTGGTAGCCCAAATC |
| dchi-R | GTCGATCAGACTATCAGCGTGAGAC |
| dcat-F | GATGGCTTCCATGTCGGCAGAATGC |
| dcat-R | GCAGCGTCTATGGCCATAGGAATAG |
| dpolh-F | CGCATCTCAACACGACTATGATAG |
| dpolh-R | CAAGTAGCGAAGCGAGCAGGACTG |
| d1629-F | GCTCTTACCAGCCTAACTTCGATC |
| d1629-R | CCTTCTTCCGTGCCTAACATTC |
| dchl-F | AATCAATTCGTTATGTTTGATTG |
| dchl-R | GGAACAGAGAACGTCACACCGTC |
