# Supplementary material for: A Highly Efficient and Simple Construction Strategy for Producing Recombinant Baculovirus Bombyx mori Nucleopolyhedrovirus
Source: PLoS One. 2016 Mar 23;11(3):e0152140. doi: 10.1371/journal.pone.0152140 (PMC4805210; doi:10.1371/journal.pone.0152140)
Supplement: S2 Table — (DOC) [file pone.0152140.s005.doc]

**S2 Table. Luciferase expression levels of recombinant BmNPV producing with different quality of viral DNA or vector DNA.**

| Parent reBmBac DNA (0.5 μg) | Transfer Vector pVL1393-luc (2 μg) | Luminescence in cells (RLU/ 50 μg protein) | Luminescence in larval haemolymph (RLU/ 50 μg protein) |
| --- | --- | --- | --- |
| prepared with L-arabinose induction | well-preserved | 8.58 ± 0.86 × 10^6^ | 3.75 ± 0.96 × 10^8^ |
| prepared with L-arabinose induction | freeze-thawed  (5 rounds) | 6.52 ± 1.07 × 10^5^ | 2.72 ± 0.50 × 10^7^ |
| prepared without L-arabinose induction | well-preserved | 2.02 ± 0.49 × 10^6^ | 6.32 ± 1.14 × 10^7^ |
